# Vaccinomics-based next-generation multi-epitope chimeric vaccine models prediction against *Leishmania tropica - a* hierarchical subtractive proteomics and immunoinformatics approach

**DOI:** 10.3389/fimmu.2023.1259612

**Published:** 2023-09-15

**Authors:** Sara Aiman, Abbas Ahmad, Azmat Ali Khan, Amer M. Alanazi, Abdus Samad, Syed Luqman Ali, Chunhua Li, Zhiguang Ren, Asifullah Khan, Saadullah Khattak

**Affiliations:** ^1^ Faculty of Environmental and Life Sciences, Beijing University of Technology, Beijing, China; ^2^ Department of Biotechnology, Abdul Wali Khan University Mardan, Mardan, Pakistan; ^3^ Pharmaceutical Biotechnology Laboratory, Department of Pharmaceutical Chemistry, College of Pharmacy, King Saud University, Riyadh, Saudi Arabia; ^4^ Department of Biochemistry, Abdul Wali Khan University Mardan (AWKUM), Mardan, Pakistan; ^5^ The First Affiliated Hospital, Henan University, Kaifeng, China; ^6^ Henan International Joint Laboratory for Nuclear Protein Regulation, School of Basic Medical Sciences, Henan University, Kaifeng, Henan, China

**Keywords:** leishmaniasis, reverse vaccinology, multi-epitope vaccine design, immunoinformatics, tropical diseases, vaccine design

## Abstract

*Leishmania tropica* is a vector-borne parasitic protozoa that is the leading cause of leishmaniasis throughout the global tropics and subtropics. *L. tropica* is a multidrug-resistant parasite with a diverse set of serological, biochemical, and genomic features. There are currently no particular vaccines available to combat leishmaniasis. The present study prioritized potential vaccine candidate proteins of *L. tropica* using subtractive proteomics and vaccinomics approaches. These vaccine candidate proteins were downstream analyzed to predict B- and T-cell epitopes based on high antigenicity, non-allergenic, and non-toxic characteristics. The top-ranked overlapping MHC-I, MHC-II, and linear B-cell epitopes were prioritized for model vaccine designing. The lead epitopes were linked together by suitable linker sequences to design multi-epitope constructs. Immunogenic adjuvant sequences were incorporated at the N-terminus of the model vaccine constructs to enhance their immunological potential. Among different combinations of constructs, four vaccine designs were selected based on their physicochemical and immunological features. The tertiary structure models of the designed vaccine constructs were predicted and verified. The molecular docking and molecular dynamic (MD) simulation analyses indicated that the vaccine design V1 demonstrated robust and stable molecular interactions with toll-like receptor 4 (TLR4). The top-ranked vaccine construct model-IV demonstrated significant expressive capability in the *E. coli* expression system during *in-silico* restriction cloning analysis. The results of the present study are intriguing; nevertheless, experimental bioassays are required to validate the efficacy of the predicted model chimeric vaccine.

## 1 Introduction

*Leishmania tropica* is a kinetoplastid parasitic protozoa and is the leading cause of leishmaniasis in tropical regions (1). The multidrug-resistant *L. tropica* is a serious health burden in the leishmaniasis endemic regions (2). *L. tropica* is a highly diverse species that covers most of Africa and Eurasia with a wide variety of serological, biochemical, and genomic characteristics (3). Leishmaniasis affects approximately 350 million people in 98 countries. Annually, around 12 million *leishmania* cases are reported worldwide, with approximately 2 to 2.5 million new occurrences. There are three main pathogenic types of leishmaniasis: mucocutaneous leishmaniasis (ML), cutaneous leishmaniasis (CL), and kala-azar (VL) (MCL). Among these CL is the most common type and affects the skin, causing scarring lesions. Approximately 1 to 1.5 million cases of CL are reported annually (4). VL is the deadliest type and is characterized by strong inflammatory reactions in the spleen and liver that may be fatal (4). A survey in 2018 revealed that more than 85% of new CL cases happened in different countries worldwide. Poverty, population shifts, malnutrition, poor hygiene, and immunocompromised conditions are the key risk factors associated with leishmaniasis (5).

The skin sores occur up to weeks to months at a site of female sandfly bite that can lead to severe conditions, if not treated. The clinical spectrum of leishmaniasis ranges from CL with varying degrees of lesion severity to VL. Several individuals have developed post-kala-azar leishmaniasis after medication, whereas nasopharyngeal MCL is uncommon (6). There is no commercially available vaccine that provides protection against leishmaniasis, although various studies have been conducted in this regard. Recombinant vaccines, such as Leish-F1, may provide some level of protection against natural infection (7). Recently, the ChAd63-KH DNA vaccine was reported promising in preventing *Leishmania* infection caused by *Leshmania donovani* and *Leshmania infantum*; however, this vaccine needs additional clinical evaluation (8). There are no reports available for commercial vaccines against *L. tropica*. Recent reports highlighted slight variations in antigen presentation as one of the possible reasons for vaccination failure in clinical trials (9).

The rapid genome sequencing technologies facilitated the availability of various pathogenic genomes. The accessibility to genome sequences of *Leishmania* species allows the researchers to detect genes involved in diseased pathways, leading to the determination of homologous antigenic peptides that could be used to develop a pan-*Leishmania* vaccine (10). A significant shift from traditional culture-based techniques to next-generation genome-based vaccine development approaches has emerged recently due to the advancement of the genomic era. It is based on genomic and immunological data to determine relevant antigenic peptides for the purpose of vaccine designing (11). The vaccine designed based on the proteome fragments of the parasite enables the vaccine to elicit humoral and cellular immune responses against the pathogen (12). The present study aimed to identify highly immunogenic B- and T-cell epitopes from lead vaccine candidate proteins to engineer a multi-peptide-based vaccine construct against *L. tropic* infection. The chimeric vaccine construct models were evaluated using immunoinformatics approaches to determine their immunogenic potential. Biophysical techniques were employed to determine structural dynamics and binding affinities of the top-ranked vaccine construct model with human immune-cell receptors.

## 2 Methodology

**Figure 1:**
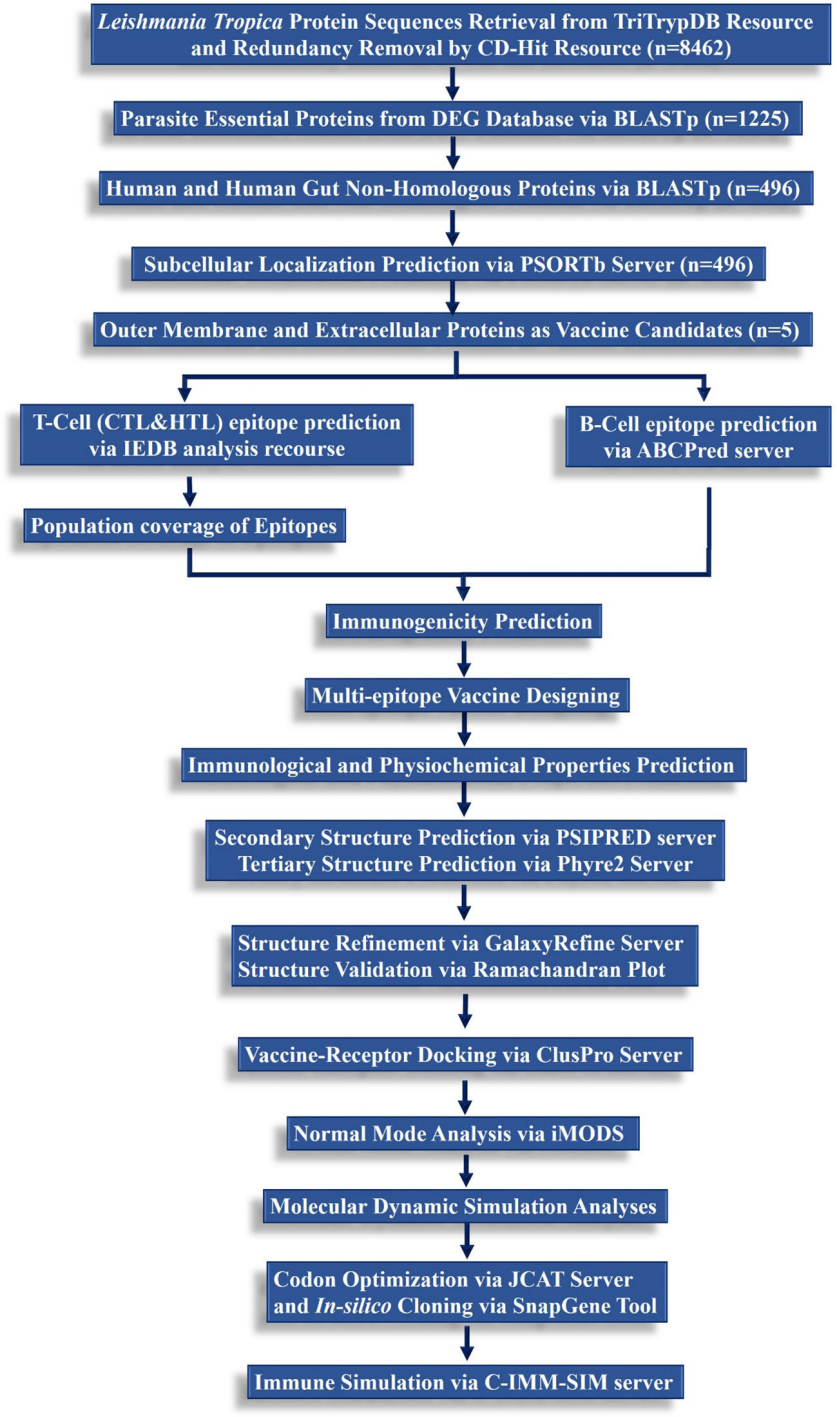
A hierarchical workflow of the present study following subtractive proteomics and reverse vaccinomics approaches to design a multi-peptide-based vaccine against *L. tropica*.

A step-wise methodological perspective of the present study is depicted in **Figure 1**.

### 2.1 Proteome sequences retrieval

Protein sequences for *L. tropica* were obtained from TriTrypDB- kinetoplastid informatics resource (https://tritrypdb.org/tritrypdb/app/) following a recently published study (13). Non-paralogous protein sequences were obtained based on a sequence similarity index threshold of 80% using the CD-HIT tool (14).

### 2.2 Identification of pathogen essential proteins

Non-redundant protein sequences were screened against the Database of Essential Genes (DEG) to identify key proteins essential for the cellular survival of the pathogen. DEG repository holds cellular essential genes from archaea, bacteria, and eukaryotes that have been experimentally verified (15). A custom database was created from essential genes of eukaryotic using the “makedb” executable of the standalone BLASTp program (16). The BLASTp parameters were set at an E-value cut-off of 10^-4^, ≥100 bit score, and ≥ 35% query coverage and sequence identity.

### 2.3 Identification of human host non-homologous proteins

The parasite-essential proteins were further subjected to BLASTp analysis against the human proteome (17) data, obtained from the NCBI database (18), to identify pathogen-essential proteins, non-homologous to host proteins. The resultant human non-homologous protein sequences of the pathogen were screened against human gut microbiota proteins (19). The BLASTp threshold criteria of bit score ≤100, sequence identity and query coverage of ≤35% and E-value cut-off of 10^-4^ were followed during this step.

### 2.4 Subcellular localization and vaccine candidates’ selection

Parasite proteins were further subjected to subcellular localization using the PSORTb v.3.0.2 web server (20). The outer membrane and extracellular proteins were prioritized for downstream investigation.

### 2.5 Prediction of CTLs, HTLs, and linear B-cell epitopes

The surface topology of the prioritized proteins is crucial for predicting the immunogenic epitopes for the chimeric vaccine construct design. Proteins in the extracellular regions were prioritized for identifying B- and T-cell epitopes. ABCPred online resource was employed to predict B-cell epitopes from the prioritized protein sequences following a set threshold of >0.5. ABCPred identifies linear B-cell peptide sequences using Support Vector Machine (SVM) and artificial intelligence approaches (21). The Immune Epitope Database (IEDB) resource was utilized to identify T-cell epitopes. CD8+ helper T lymphocytes (HTL) are MHC-I-restricted epitopes that were determined using the Stabilized Scoring Method (SSM). MHC-II epitopes i.e., CD4+ cytotoxic T lymphocytes (CTL), were determined using the neural network-based scoring method (NetMHC 1.1). The human HLA reference alleles set was utilized with the IC_50_ <200 nM (22). Lead CTL, HTL, and linear B-cell epitopes were further prioritized based on immunogenic properties. The antigenic behavior of the prioritized epitopes was evaluated using the VaxiJen v2.0 web tool with a cut-off of 0.4 (23). The allergenicity of the epitopes was determined by AllerTOP 2.0 tool (24) and the toxicity was determined by the ToxinPred server (25).

### 2.6 Assessment of population coverage

Variations in the distribution and expression of HLA alleles among various ethnic groups and geographic regions around the world strongly impact the effectiveness of vaccine synthesis. Therefore, the global population coverage of the prioritized CTL and HTL epitopes and their respective HLA-binding alleles was determined using the IEDB population coverage tool (26). Conservation analysis is important to measure the degree of similarity between homologous peptides.

### 2.7 Multi-epitope chimeric vaccine designing

The top-ranked overlapped CTL, HTL, and linear B-cell epitopes of 9-20 mer were prioritized based on their antigenicity, allergenicity, and toxicity values to design chimeric vaccine models. The rationale behind the selection of overlapped epitopes was to induce both cell-mediated and humoral immune responses against the immunogenic antigen (27). Besides potential immunostimulatory adjuvants were used to induce enhanced immune responses and to initiate long-term innate immunity (28). Four different adjuvant sequences, i.e. HBHA protein, β-defensin, 50S ribosomal protein L7/L12, and HBHA conserved peptide sequence were used in designing the constructs. EAAAK linker (stiff spacer) was used to connect the adjuvant sequence to the N-terminus of the model construct (29). GGGS and HEYGAEALERG flexible linkers were used for effective epitope conjugation based on Solanki & Tiwari’s (30) approach. PADRE sequences were also incorporated to enhance the vaccine construct stability and induce innate and adaptive immune responses. The linkers prevent junctional epitope development, enhance the expression and bioactivity of the vaccine construct, and stimulate robust immunogenic responses against the antigenic vaccine (30).

### 2.8 Physicochemical and immunological assessment of model vaccine constructs

The chimeric vaccine constructs were evaluated by following the physicochemical and immunological parameters. ExPASy ProtParam web tool was utilized to evaluate multiple physicochemical properties of the constructs (31). The solubility feature was calculated using the SOLpro web tool (32). The antigenicity, allergenicity, and toxicity evaluation of the vaccine constructs was conducted by Vaxijen v2.0 (23), AllerTOP2.0 (24), and ToxinPred2 (25) web servers, respectively. ANTIGENpro web tool evaluated the antigenic nature of the designed vaccine constructs. The tool uses 10-fold cross-validation of the antigenic peptides to calculate the protective capabilities of the peptide sequences based on known datasets (33).

### 2.9 Assessment of secondary and tertiary structures

PSIPRED v 4.0 (34) and SOPMA (35) tools were used to analyze and assess the secondary structure and composition quality aspects of the model vaccine constructs. These servers use a Position-Specific Scoring Matrix (PSSM) to predict transmembrane topologies, transmembrane helices, and peptide sequences with folds and domains. The tertiary structure of vaccine constructs was predicted using the Phyre2 web tool following 99% structure confidence parameter (36). The predicted 3D structures of the designed vaccine constructs were further refined using the GalaxyRefine tool. GalaxyRefine uses CASP10-based refinement approaches to achieve a relaxed protein structure based on repacking and molecular dynamic modeling techniques. This approach improves the quality of predicted protein models and enhance global and local structural features (37). After refinement, the 3D structures of the designed vaccines were validated by ERRAT, PROCHECK (38), and ProSA-web (39) servers. ProSA-web server determines the overall quality of the vaccine structure by calculating the Z-score. The Z-score value beyond the threshold established for natural proteins indicates errors in the protein model (39). PROCHECK tool determines the geometry of the residues in protein structure and generates a Ramachandran plot to evaluate the stereochemical quality of the designed vaccine models.

### 2.10 Determination of discontinuous B-cell epitopes

Linear B-cell epitope determination is dependent upon the amino acid sequence of the designed vaccine. Nevertheless, certain epitopes are also conformational/discontinuous, relying on the 3D structure of the model vaccine rather than the linear sequence (40). The refined 3D structure of the model vaccine construct was subjected to the ElliPro web server to determine discontinuous B-cell epitopes. The server is based on residue clustering and Tornton’s methods for discontinuous B-cell epitopes prediction and assigns PI (protrusion index) value to the predicted epitopes (40).

### 2.11 Molecular docking with host immune receptor

Molecular docking provides an insight into the binding interaction between the model vaccine constructs and the immune receptor proteins. The molecular docking study of the designed vaccine constructs with the human toll-like receptors 4 (TLR4) was conducted using the ClusPro resource. ClusPro is a robust and highly integrated protein-protein docking server that incorporates a hybrid docking algorithm based on experimental data of substrate binding site of protein and small-angle X-ray scattering to perform docking analyses to generate 10 models (41). The refined 3D structure of the vaccine construct (ligand) was docked against TLR4 (PDB:3FXI) receptor. The molecular interactions between the residues in the vaccine-TLR4 complex were demonstrated via the PDBsum (42).

### 2.12 Normal mode analysis

The normal mode analysis (NMA) was conducted for the finalized V1-TLR4 complex using the iMODS server (43) to demonstrate the internal dihedral coordinates and assess the cooperative functional motions of the peptide vaccine. The essential dynamic simulation program of iMODS was used to ensure the energy minimization, molecular stability, and atomic mobility of the prioritized vaccine construct in the docked complex. The server calculates the probable motions of the V1-TLR4 complex based on specialized parameters, including B-factors, eigenvalues, RMSD, deformability of the complex, covariance values, and elastic models (43).

### 2.13 Molecular dynamic simulation analyses

#### 2.13.1 Desmond MD simulations

The MD simulations were performed to study the dynamics and stability of the vaccine-TLR4 docking complex using the Desmond program of Schrödinger suite 2023-1 (Schrödinger, LLC, New York, NY, USA) (44). The coordinate file and topology of the vaccine-receptor complex were generated using the Optimized Potentials for Liquid Simulation (OPLS4) force field in the initial step (45). The complex was further solvated in transferable intermolecular potential 3P (TIP3P) by adding a hydrated box with a boundary size of 10 Å (46), followed by counter ions (Na^+^ and Cl^-^) to neutralize the charges in the simulated system. Furthermore, energy minimization was carried out to acquire the desired threshold of ≥1000.0 kJ/mol/nm. NVT and NPT simulations were conducted for 1 ns, respectively, to generate a stable temperature and pressure environment. The temperature of the simulated system was equilibrated to 310 K and pressure was maintained at 1 atm. The Berendsen thermostat was used to regulate the temperature, and the Parinello-Rahman barostat was employed to equilibrate the pressure of the system. Finally, the hydrogen bonds were constrained using the LINCS technique, and the long-range electrostatic interactions were calculated using the Particle-Mesh Ewald summation scheme (47). The MD simulation was run with an isothermal and isobaric system for 200 ns.

#### 2.13.2 Principal component analysis

The principal component analysis (PCA) was performed to detect the high amplitude primary movements and conformation changes from the MD trajectory. The principal components (PCs) are eigenvectors that identify the direction of motion, and the corresponding eigenvalues describe the degree of residual motion during the MD. Using the ProDy package, the covariance matrix was first measured by using the Cα coordinates, and then the covariance matrix was diagonalized to construct the eigenvalues and eigenvectors (PCs) (48). The mode vectors were visualized using VMD (Visual Molecular Dynamics) software to understand the relationship between the movements of the vaccine construct and the motions of TLR4 residues.

### 2.14 Computational immune simulations of model vaccine construct

The C-ImmSim web application was followed to perform computational immunological simulation of the top-prioritized designed vaccine model (49). The server computes cellular and humoral responses produced against the antigenic vaccine using PSSM and several machine-learning algorithms (50). The server uses antigenic peptide sequences and lymphocyte receptors to simulate the immunogenic responses. In this study, a standard clinical protocol of a four-week period between two doses was followed to conduct an immune simulation of the designed vaccine construct (51). The human host leukocyte antigens HLA-A*0101, HLA-A*0201, HLA-B*0702, HLA-B*3901, HLA-DRB1*0101, and HLA-DRB1*0401 were selected for time periods of 1h, 84h, and 168h. Immune simulation was performed using default settings for 1000 steps (29).

### 2.15 Computational cloning assessment of optimized vaccine construct sequence

The reverse translation of the prioritized vaccine model was performed using the Java Codon Adaptation Tool (JCAT). Codons in the cDNA sequence of the construct were optimized to maximize the model vaccine’s expression in the bacterial expression system (52). JCAT calculates codon adaptation index (CAI) and percentage CG content to assess the protein expression capability of the construct. The optimal CAI value is stated to be 0.8-1 and the GC content should range from 30 to 70% for promising transcriptional and translational efficacies of vaccine (29, 53). The *E. coli* strain K12 was chosen as the host organism for the expression of the model vaccine. *E. coli* strain K12 was selected because it is commonly used to clone human vaccines in mouse models against many pathogens and highly protective antibody production has been evident in experimental models using this strain (54). *E. coli* pET28(+) vector was retrieved from the Addgene server (55) for *in-silico* restriction cloning. The SnapGene program (https://www.snapgene.com/) was used to introduce the optimized construct sequence of the model vaccine in *E. coli* plasmid.

## 3 Results

### 3.1 Subtractive proteomics analysis

In the current study, we employed a subtractive proteomics strategy to determine parasite-specific vaccine candidate proteins to design multi-epitope vaccine constructs. A total of 8462 non-paralogous protein sequences of the *L. tropica* strain L590 were obtained from CD-Hit clustering recourse with a calling criterion of 0.8 (**Supplementary File 1**). Non-paralogous protein sequences were subjected to the DEG database and analysis identified a total of 1225 parasite essential proteins (**Supplementary File 2**). These proteins are responsible for the survival of the parasite and may be involved in pathogenesis. A total of 496 host non-homologous parasite-essential proteins were identified after screening against human and human gut proteomes (**Supplementary File 3**).

### 3.2 Subcellular localization and vaccine candidate selection

The parasite-essential proteins that were non-homologous to human and human gut proteomes, were prioritized for subcellular localization prediction. Analysis categorized proteins into cytoplasm, cytoplasmic membrane, extracellular, and cell wall localization (**Table 1**). Based on surface topology, five proteins i.e., LTRL590_220014100, LTRL590_090006100, LTRL590_100005200, LTRL590_180011200, and LTRL590_190013800 located in the extracellular region were prioritized and screened for B- and T-cell epitopes identification. LTRL590_220014100 is a conserved hypothetical protein predicted in the extracellular region of the parasite. LTRL590_090006100 is an RNA-binding 5-like protein that is associated with the post-transcriptional modulation of the parasite proteins and plays a major role in parasite survival and adaptation to the drastic environmental change from amastigotes into promastigotes (56). The LTRL590_100005200 is a putative helicase protein that is associated with cellular DNA repair pathways and maintains genome stability throughout the differentiation from amastigotes into promastigotes stages in the leishmania cycle, hence plays a crucial role in the survival of the pathogen (57). The putative FYVE zinc finger containing protein (LTRL590_190013800) plays a major role in the virulence of the *leishmania* parasite by mediating protein-protein interactions and membrane association (58). These proteins also bind to nucleic acids and play an essential role in transcription, translation, and other biological processes during the transition from amastigotes to promastigotes (59).

**Table 1:** Subcellular localization of host non-homologous essential proteins of *L. tropica*.

Subcellular localization	Number of proteins
Cytoplasm	264
Unknown	125
Cytoplasmic membrane	94
Extracellular	5
Cell wall	8

### 3.3 Lead B- and T-cell epitopes prediction

**Figure 2:**
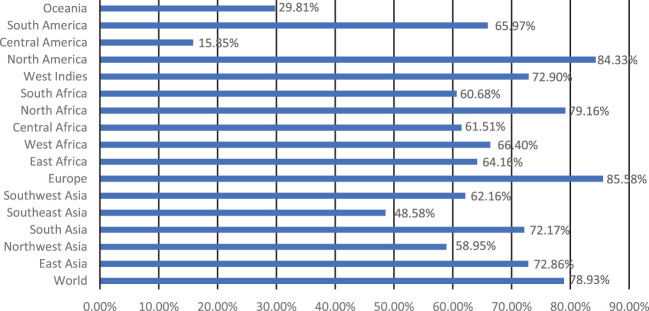
Population coverage of the prioritized MHC epitopes determined by IEDB analysis resource.

Five extracellular vaccine candidate proteins were prioritized to predict B and T-cell epitopes. These epitopes were ranked based on high antigenicity and low allergenicity, and toxicity values. Only two CTL and HTL epitopes of 9-15-mer were prioritized from each vaccine candidate protein based on an IC_50_ <200 nM using the IEDB analysis resource. Likewise, two linear B-cell epitopes of 20-mer were prioritized from each prioritized protein. The top-ranked antigenic, non-allergenic, and non-toxic linear B-cell, CTL, and HTL epitopes sharing overlap peptides were prioritized for engineering model chimeric vaccine constructs (**Table 2**). The prioritized MHC epitopes exhibited population coverage of 79% approximately. North America was found as the largest population coverage of MHC epitopes (84.33%). The tropical and sub-tropical regions exhibited the highest density of these epitopes (**Figure 2**). Therefore, the vaccine designed based on these epitopes will provide significant protection against leishmaniasis in these regions.

**Table 2:** Prioritized B- and T-cell epitopes predicted by ABCPred and IEDB web servers.

Gene IDs	B-cell Epitopes	Score	MHC-I Epitopes	Alleles	IC_50_	MHC-II Epitopes	Alleles	IC_50_
**LTRL590_220014100**	MSLPNWSANRQSIGARAGPA	0.83	MSLPNWSANR	HLA-A*31:01 HLA-A*68:01 HLA-A*33:01	21.93	WGQLELDMSLPNWSA	HLA-DRB1*03:01 HLA-DRB3*01:01	171
	YTLDAAVCVPFGGSGADASR	0.87	YTLDAAVCV	HLA-A*02:06 HLA-A*02:01 HLA-A*68:02 HLA-A*02:06	17.17	HRYQYTLDAAVCVPF	HLA-DRB3*01:01 HLA-DRB3*02:02 HLA-DRB1*07:01	5.5
**LTRL590_090006100**	FFTPFGVTAGTESDLMASAL	0.87	SPAPAFFTPF	HLA-B*07:02 HLA-B*35:01 HLA-B*15:01	22.49	PAFFTPFGVTAGTES	HLA-DRB1*07:01	147.9
	RASSSNVTPINYSAHVVPSQ	0.85	STRASSSNV	HLA-A*30:01	7.35	GTFTESPGSTRASSS	HLA-DRB5*01:01	159.6
**LTRL590_100005200**	TLKRDERDDASGRDTRNLSF	0.88	FETLKRDERD	HLA-B*07:02	13.26	AVKRFYETLKRDERD	HLA-DRB5*01:01	38.2
	SRTQIPLRHAWALTIHKSQG	0.82	ASRTQIPLR	HLA-A*31:01 HLA-A*30:01 HLA-A*68:01 HLA-A*31:01	20.22	GIMVSSAASSASRTQ	HLA-DRB3*02:02	20.5
**LTRL590_180011200**	NGGAPSSTSVATVYASPTQA	0.88	TVYASPTQA	HLA-A*02:03 HLA-A*68:02	87.42	QVVYTASPTQAAGVM	HLA-DRB5*01:01	169.39
	PTPTQMLTPQANAAAAAAAV	0.86	TPAQPTPTQM	HLA-B*07:02 HLA-B*35:01	44.1	YTPDAFPTPTQMAAA	HLA-DRB1*07:01	181.26
**LTRL590_190013800**	YVAAPQDTGRASVGVEHRVI	0.88	KQDTSYVAA	HLA-A*02:06	19	SADYVAAAPGYSAHT	HLA-DRB5*01:01	118
	VVSNELAVSHKTCGREEAAT	0.88	VVSNELAVSH	HLA-A*33:01	195	VVSNELAVSRRRVLC	HLA-DRB4*01:01	33.18

The red color indicates the overlapping residues in the MHC-I, MHC-II, and B-cell epitopes.

### 3.4 Designing multi-epitope chimeric vaccine models

The overlapped lead CTL, HTL, and B-cell epitopes were used to engineer a highly immunogenic multi-epitope chimeric vaccine with the capability to elicit significant immune responses against *L. tropica*. Ten overlapping epitopes were selected and linked by GGGS and HEYGAEALERG linkers to design such a construct. Four different adjuvants were attached at the N-terminal of the multi-epitope peptide sequence using EAAAK linkers to design four different vaccine constructs. The vaccine construct-V1 was designed using HBHA adjuvant. Likewise, the V2, V3, and V4 constructs were designed using β-definsin, HBHA conserved protein, and ribosomal protein adjuvants respectively (**Supplementary Data**). PADRE peptides were inserted in the peptide sequence of the designed vaccine construct to overcome HLA-DR challenges and enhance immune protection and HLA responses (60). The proposed vaccine constructs were designed to stimulate specific immune responses with reduced risk of antigen-induced anaphylaxis.

The immunological properties determined all four of the designed vaccine constructs to be highly antigenic, non-toxic, and non-allergenic in nature. VaxiJen v2.0 scores for all the constructs ranged from 0.96 to 1.15, which is above the set antigenic threshold. The estimated range of ANTIGENpro scores ranged from ~0.85 to ~0.95, indicating the highest antigenicity of the vaccine constructs. The estimated molecular weights of the designed vaccine constructs ranged from 36 to 48 kDa, indicating the ideal molecular weight for commercial production. The SOLpro values ranged from ~0.57 to ~0.60, suggesting high solubility of the vaccine constructs. The estimated GRAVY values were in the range of -0.068 and -0.278, suggesting the hydrophilic nature of the model constructs. The theoretical pI scores ranged from 5.59 to 9.63, the aliphatic index ranged from 62.79 to 74.27, and the instability index values ranged from 32.29 to 43.00. These results indicate the thermostability of the model vaccine constructs at different temperature ranges. All of the model vaccine designs were non-allergenic, non-toxic, and highly antigenic due to their physicochemical and immunological features. The stringent threshold of physicochemical and immunological characteristics predicted that the proposed constructs depict thermostability and substantial abilities to stimulate strong immunogenic responses against leishmaniasis (**Tables 3** and **4**).

**Table 3:** Physicochemical properties, % GC content, and CAI values of the model vaccine constructs calculated by ProtParam and JCAT tools.

Vaccine construct	Adjuvants	Number of Amino Acids	Molecular Weight (Daltons)	Theoretical pI	Aliphatic index	Grand average of hydropathicity (GRAVY)	Instability index	GC content	CAI
V1	HBHA	476	48kDa	5.81	71.83	-0.278	39.59	50%	1.0
V2	β-defensin	362	36kDa	9.63	62.79	-0.235	38.31	50.73%	1.0
V3	HBHA conserved	567	47kDa	5.59	73.81	-0.253	43.00	49.73%	1.0
V4	Ribosomal protein	457	44kDa	5.91	74.27	-0.068	32.29	48.83%	1.0

**Table 4:** Immunological properties and docking scores of the designed vaccine constructs.

Vaccine construct	Allergenicity	Antigenicity (Vaxijen 2.0 scores)	ANTIGENpro Scores	SOLpro scores	Docking score against TLR4 (kcal/mol)
V1	Non-Allergen	0.9952	0.886112	0.576670	-886.7
V2	Non-Allergen	1.1411	0.948980	0.599276	-869.1
V3	Non-Allergen	1.0045	0.844777	0.607072	-882.3
V4	Non-Allergen	0.9685	0.913701	0.570929	-592.9

### 3.5 Secondary and tertiary structures prediction, refinement, and validation

**Figure 3:**
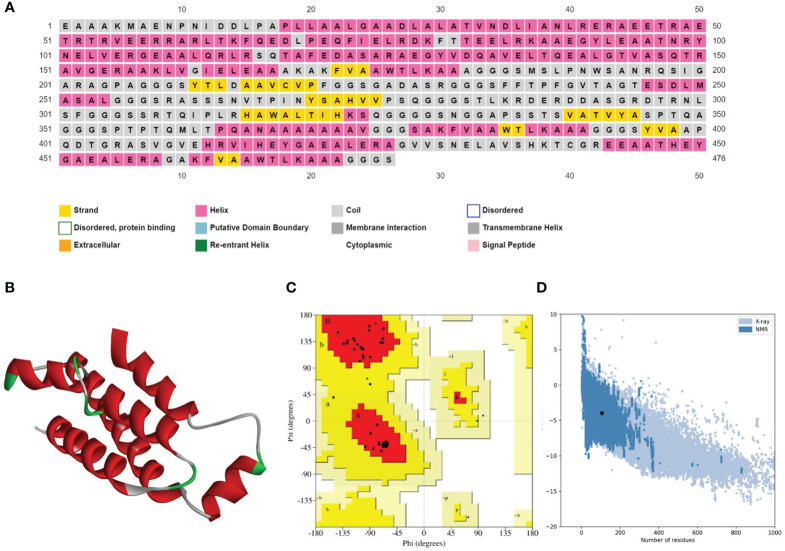
**(A)** Secondary structure prediction of the prioritized vaccine construct-V1 acquired from the PSIPRED server revealed that the majority of the structure comprises of α-helices and coils. **(B)** Refined 3D structure of the prioritized vaccine construct-V1. **(C)** Ramachandran plot of the prioritized vaccine construct-V1 showing model accuracy **(D)** ProSA-web results of the prioritized vaccine construct-V1 show a Z-score of -3.95, representing stable tertiary structure with minimum errors.

The secondary structure prediction analysis unraveled that the designed vaccine constructs (V1-V4) comprise ~32% to 52% α-helices, ~5% to 10% β-stands, ~29% to 43% random coils, and ~11% to 18% extended strains. The tertiary structure prediction of all four vaccine constructs was performed using a homology-based strategy. The modeled structures were further refined to generate high-quality 3D structure models. Ramachandran plot analysis depicted that ~91 to 99% of residues of the refined vaccine structure models occurred in the core region of the plot. ERRAT values were calculated to be in the range of ~97 to 100 and the Z-scores for the proposed vaccine models were estimated between -3 to -7 (**Table 5**). These results suggest that the designed vaccine construct models were of high quality with significant 3D structural stability. The secondary and tertiary structures of the top prioritized model vaccine-V1 have been shown in **Figure 3**.

**Table 5:** Secondary and tertiary structures prediction and validation of the designed vaccine constructs.

Vaccine Constructs	Alpha Helices	Extended strand	Beta turns	Random coil	ERRAT	PROCHECK Ramachandran core region	ProSA-Web Z-score
V1	51.89%	11.13%	5.67%	31.30%	97.9592	94.0%	-3.95
V2	32.04%	17.13%	8.56%	42.47%	100	86.8%	-4.61
V3	53.10%	11.35%	5.78%	29.76%	98.2759	91.6%	-3.33
V4	43.62%	14.32%	9.17%	32.89%	100	98.3%	-7.25

### 3.6 Discontinuous B-cell epitopes prediction

**Figure 4:**
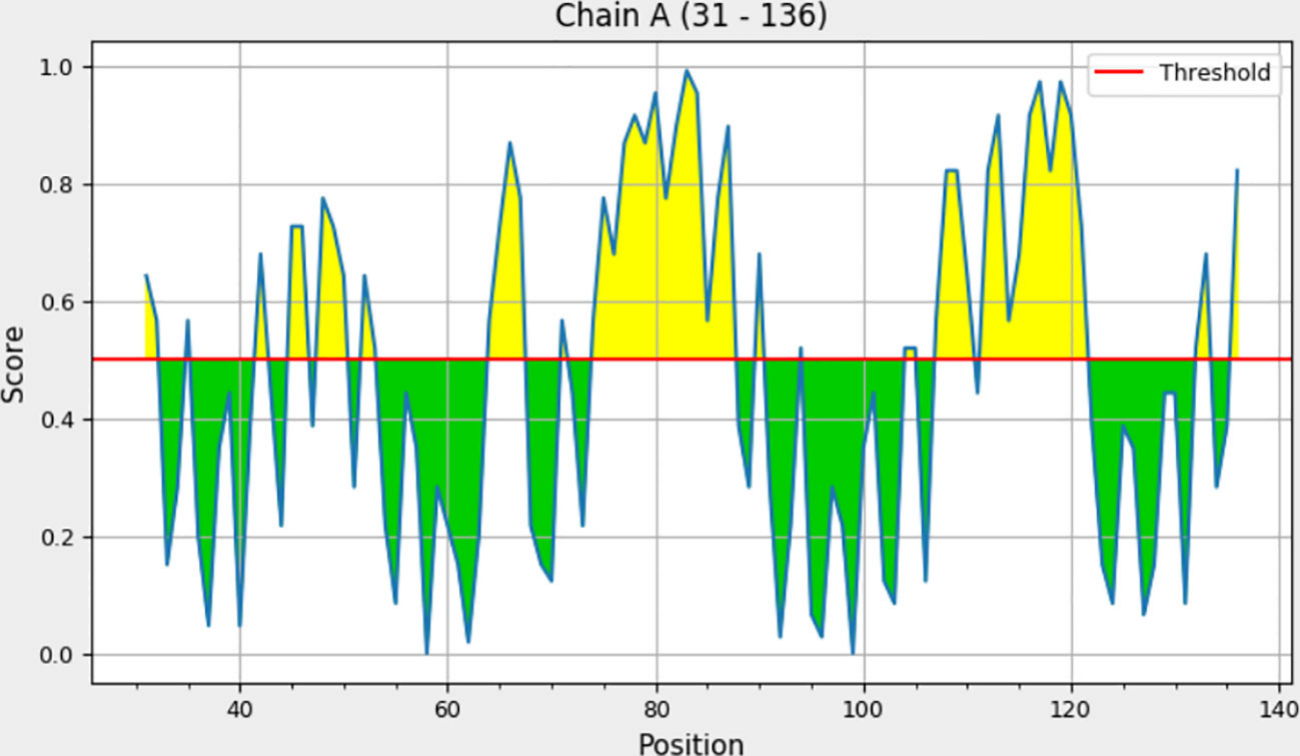
ElliPro residue score chart for each discontinuous B-cell epitope with a score above 0.6.

Explicit interactions occur between B-cells and the epitopes at a 3D level in their folded state. Therefore, discontinuous/conformational epitopes with various residues were determined from the 3D structure of the finalized vaccine construct. The ElliPro web tool identified 4 conformational B-cell epitopes at a threshold of 0.5 and scores varied from 0.618 to 0.855. The size of the epitopes ranged from 6 to 14 amino acid residues (**Table 6** and **Figure 4**).

**Table 6:** Discontinuous B-cell epitopes predicted by ElliPro web tool.

No.	Residues	Number of residues	Score
1	A:F80, A:T81, A:T82, A:E83, A:E84, A:L85	6	0.855
2	A:G107, A:E108, A:A109, A:A110, A:Q112, A:R113, A:L114, A:R115, A:S116, A:Q117, A:T118, A:A119, A:F120, A:E121	14	0.796
3	A:Q66, A:D68, A:P70, A:E71, A:I74, A:E75, A:L76, A:R77, A:D78, A:K79	10	0.644
4	A:E42, A:E45, A:E46, A:T47, A:R48, A:A49, A:E50, A:T51	8	0.618

### 3.7 Vaccine-TLR4 docking analysis

**Figure 5:**
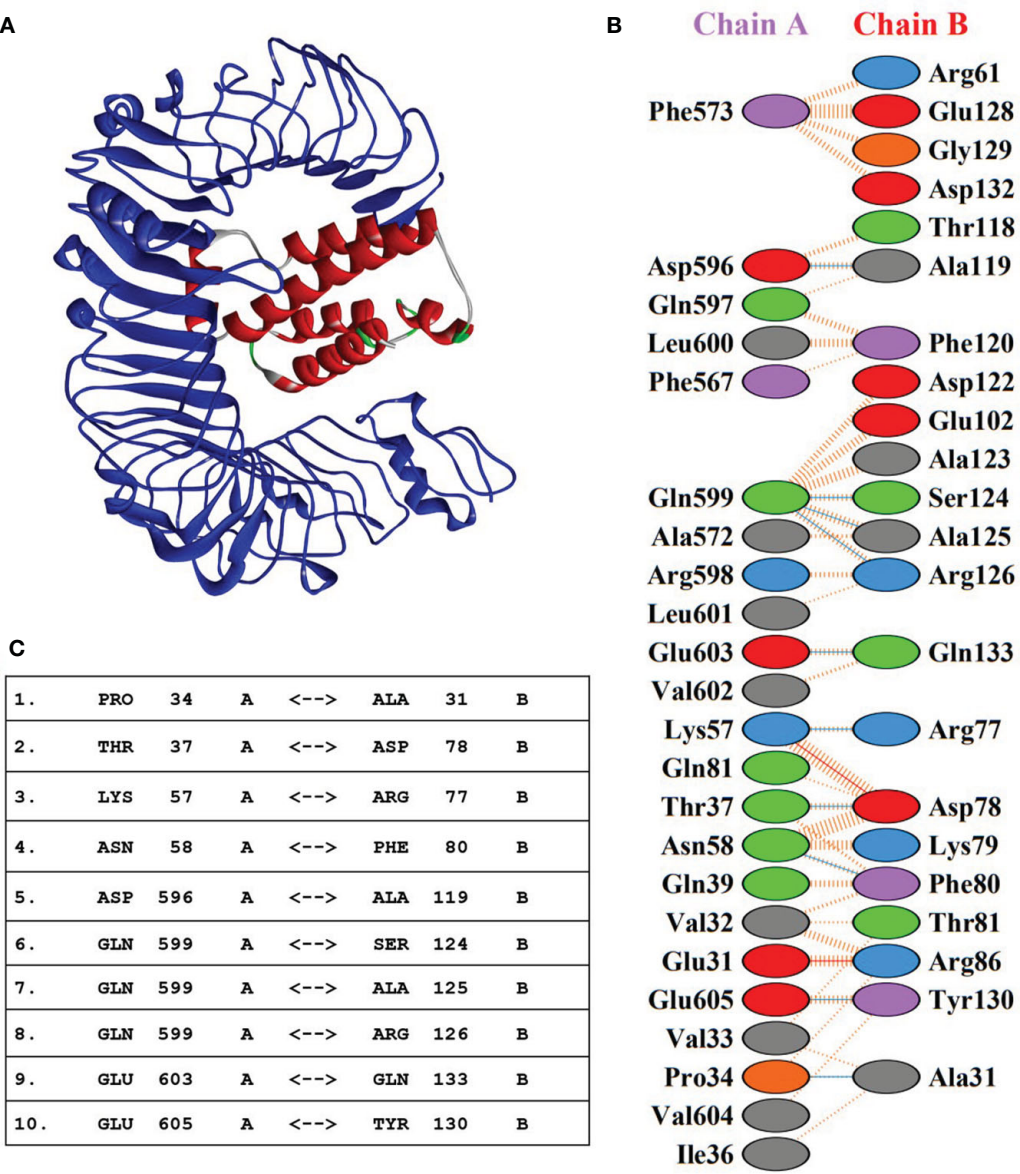
**(A)** 3D representation of the docking complex of vaccine construct V1 (red) with human TLR4 receptor (blue). **(B)** Molecular interactions between chain-A of TLR4 receptor molecule and chain-B of vaccine construct-V1. **(C)** Hydrogen-bond interaction between chain-A of TLR4 receptor molecule and chain-B of vaccine construct-V1.

Molecular interaction between the vaccine construct and human immune receptor is crucial for understanding the antigenic potential of the designed vaccine construct to elicit immunogenic responses. TLR4 immune receptor is involved in recognizing pathogenic proteins, which in turn triggers the generation of inflammatory cytokines. Numerous studies have reported that TLR4 is essential for generating innate immune responses against an array of infections (61, 62). The molecular docking studies were carried out to evaluate the molecular interactions between vaccine constructs. All four vaccine constructs were docked against the human TLR4 receptor. The vaccine construct-V1 in complex with TLR4 exhibited the lowest binding energy of -886.7 kcal/mol. (**Figure 5A** and **Table 5**). The lowest binding energy measured the highest binding affinity between the vaccine construct-V1 and the TLR4 receptor. A feasible molecular interaction was found among vaccine construct-V1 and TLR4 receptor residues (**Figure 5B**). A total of two salt bridges, ten hydrogen-bond interactions (**Figure 5C**), and 151 non-bonding interactions were observed between the construct-V1-TLR4 complex chains. These results signified the ability of the vaccine construct to stably bind with immune receptors and induce an immunological response in the human immune system against leishmaniasis.

### 3.8 Normal mode analysis

**Figure 6:**
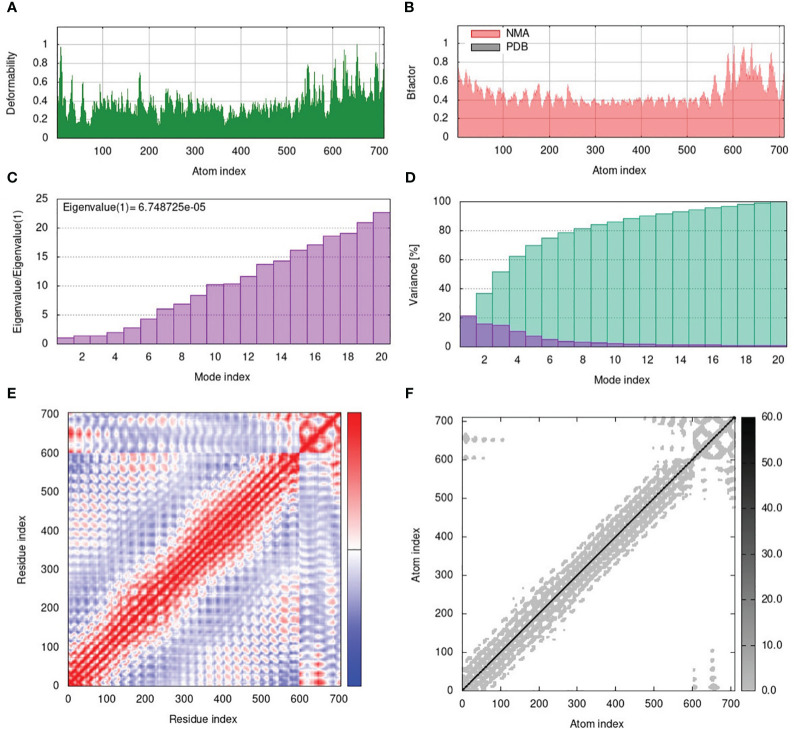
**(A)** The main-chain deformability of the V1-TLR4 complex deformed at each of its residues showing the location of the ‘hinges/linkers’ in the peptide chain. **(B)** The B-factor represents the associated PDB field and NMA mobility in the V1-TLR4 complex. **(C)** The eigenvalue specifies the motion stiffness linked to each normal mode. **(D)** The variance map is associated with individual (red) and cumulative (green) variances. **(E)** The covariance graph represents correlated (red), uncorrelated (white), or anti-correlated (blue) mobility of the pairs of residues in the V1-TLR4 complex. **(F)** The elastic network model describes the pairs of atoms connected by springs where the darker grey dots indicate the stiffness of the springs.

The normal mode analysis (NMA) was conducted to establish the molecular stability and functional motions of the construct-V1-TLR4 complex (**Figure 6**). The deformability graph showed peak points that represent the main chain residues deformed regions in the V1-TLR4 complex. The high deformability regions can be used to determine the ‘hinges/linkers’ in the main chain (**Figure 6A**). The experimental B-factor plot demonstrates the association among the NMA mobility and V1-TLR4 complex, representing the average RMSD values of the docked complex (**Figure 6B**). The computed eigenvalue of the V1-TLR4 complex was 6.748725e-05 which reflects the motion stiffness linked to each normal mode (**Figure 6C**). Each normal mode of the complex is represented by an individual (purple) and cumulative (green) variance in the variance bar. Variance and eigenvalue were negatively correlated (**Figure 6D**). Additionally, the interacting motions between the two molecules in a complex are represented by a covariance map. In the current study, interrelated motions among different pairs of residues were specified by correlated (red), uncorrelated (white), and anti-correlated (blue) atomic motions in the V1-TLR4 complex (**Figure 6E**). The specialized electric network map was also generated which represents pair of atoms connected by spring in the V1-TLR4 complex. The assembly between corresponding atoms of larger molecules and their stiffness are represented by colored dots, where the darker greys represent rigid springs (**Figure 6F**). Eventually, the NMA analysis anticipated stable interaction between the TLR4 and the prioritized vaccine construct-V1.

### 3.9 Molecular dynamic simulation analyses

#### 3.9.1 Dynamics stability and residual flexibility of modeled vaccine–TLR4 complex

**Figure 7:**
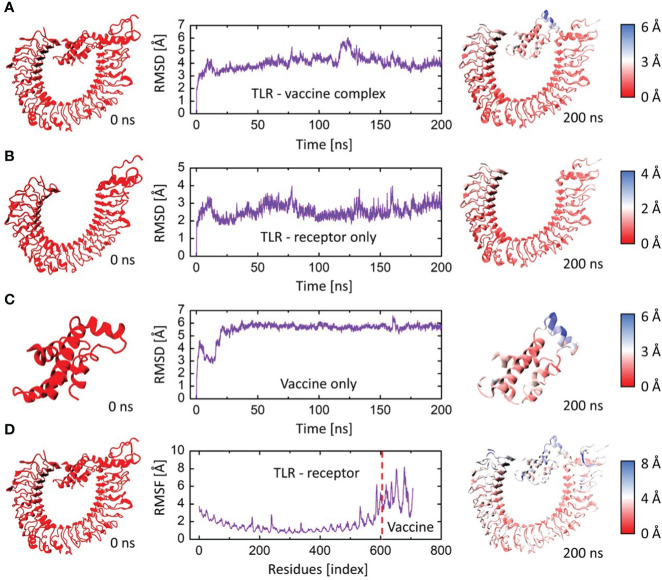
The RMSD and RMSF plots for TLR4 and vaccine construct complex. **(A)** RMSD plot of the vaccine-TLR4 complex. **(B)** RMSD plot of TLR4 receptor. **(C)** RMSD plot of vaccine construct. **(D)** RMSF plots for the vaccine-TLR4 complex. The left panel indicates the initial structure of the moieties, and the rightmost panel indicates the final conformation of the moieties. The colors are based on the Cα deviation based on the scales given for each representation.

The root mean square deviation (RMSD) in the bound and unbound state of the vaccine construct and TLR4 receptor were calculated and presented as a graph based on the Cα atoms of the protein to explain the dynamic behavior and conformational stability from initial structure conformation to its final state (**Figure 7A**). Minor deviations faced by a system in the RMSD curved indicate a stable complex formation and vice versa; for the bound state, i.e., vaccine construct in complex with TLR4 receptor. The average RMSD of 4.03 Å depicts no significant fluctuation after convergence for the entire period of simulation except minor fluctuations at 20 and 125 ns. Afterward, the system was fully converged with a standard deviation of 0.54. Furthermore, the individual chains of the TLR4 receptor and vaccine construct revealed the mean RMSD of 2.57 Å and 5.49 Å (**Figures 7B, C**), with a standard deviation of 0.38 and 0.70, respectively. The highly deviated residues based on Cα is represented in the right panel of the figure which shows that only a few residues of the vaccine fluctuated to reorient itself. This is more probably due to the strain energy upon binding with TLR4. The rest of the vaccine remained stable and predicted to interact strongly with the receptor throughout the production run. In order to further comprehend the stability of the complex formation we carried out the residual flexibility analysis which indicates the TLR4 receptor has a lower flexibility value at the binding interface i.e., Pro34, Thr37, Lys57, Asn58, Asp596, and Gln599 (**Figure 7D**) which is congruent to our previous analysis. Overall, these findings demonstrated that vaccine construct in complex with TLR4 receptor showed significant dynamic stability for the entire 200 ns of the simulation period.

#### 3.9.2 Principal component analysis

**Figure 8:**
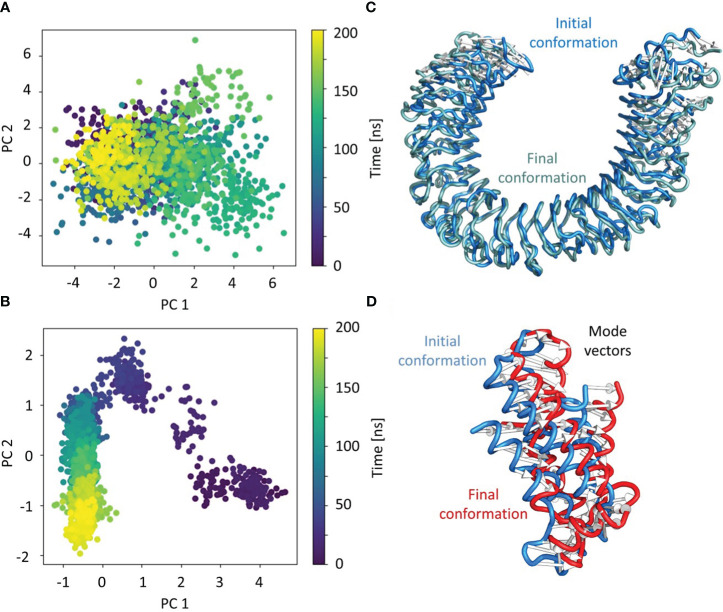
Individual principal component analysis of the conformational shifts in **(A)** TLR4 receptor, **(B)** the movements of the first principal component of the receptor from initial (cyan) to final (grey), **(C)** the vaccine construct, and **(D)** the movements of the first principal component of the vaccine construct from initial (cyan) to final (grey). The PC’s color represents the progression through the trajectory from 0 ns (dark blue) to 200 ns (yellow).

PCA was used to identify the predominant movements in TLR4 and vaccine construct-V1, and the majority of the combined dominating motions were represented by the first five eigenvectors. The first five eigenvectors for each system had significant variations, whereas subsequent eigenvectors displayed minor changes in amplitude, suggesting that the first five eigenvectors primarily represented the dynamic structural information. Herein the vaccine construct-V1 and TLR4’s first two eigenvectors i.e., PC1 and PC2, were projected against one another to display potential attributed movements. The continuous color representation from deep blue to yellow shows the periodic transition between conformations. Each dot depicts the conformation of each frame; in the case of the TLR receptor, a constant frame overlapping was observed (**Figures 8A, C**). In contrast, the vaccine construct initially underwent fluctuation at the start of the simulation due to the strain energy; however, it did not exhibit any significant internal movements after 40ns, which further supported the RMSD results (**Figures 8B, D**).

### 3.10 Immune simulations

**Figure 9:**
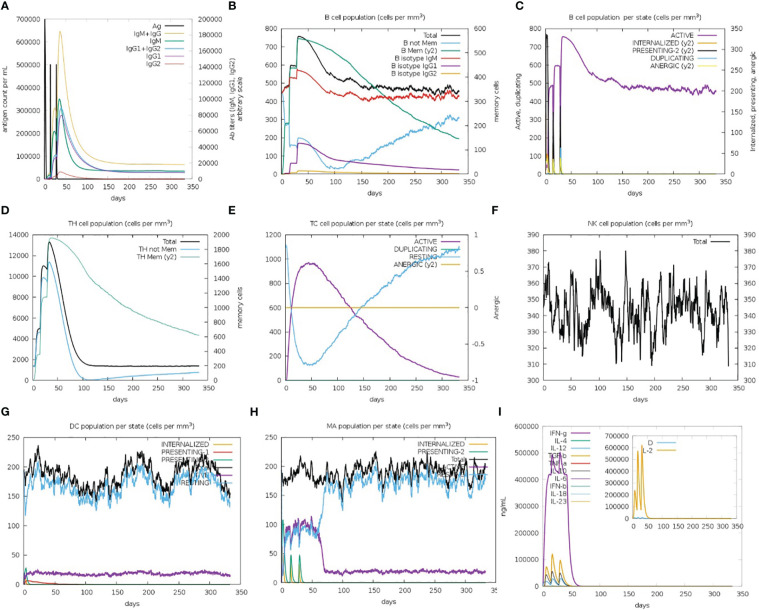
**(A)** Immunoglobin antibodies production after vaccination, representing proliferation of immune responses against the antigen **(B, C)** B-cell population increased after vaccine administration and development of memory B-cells. **(D, E)** The population of helper and cytotoxic T cell increase with a decrease in the antigen population and development of memory cell. **(F–H)** Development of natural killer cells, dendritic cells, and macrophages. **(I)** High levels of cytokines and interleukins after continuous antigen exposure.

The immune simulation results showed a significant increase in primary and secondary immune responses against the top-ranked proposed vaccine construct-V1 (**Figure 9**). High levels of IgG1 + IgG2, IgM, and IgM + IgG immunoglobin antibodies were observed after the vaccine administration, representing proliferation of immune responses (**Figure 9A**). Increase in the B-cell population was evident after repeated exposure of the antigenic vaccine, resulting in the development of humoral immune memory (**Figures 9B, C**). The population of cytotoxic and helper T cells increased with a substantial decrease in the antigen population during secondary and tertiary immune responses (**Figures 9D, E**). This indicates the enhanced adaptive immunity capability of the proposed vaccine model (63, 64). Moreover, the development of natural killer cells, dendritic cells, and macrophages was also predicted to sustain growth after each immunization (**Figures 9F–H**). Vaccine dosages are commonly reported to stimulate the release of a variety of cytokines, including IFN-gamma, IL-23 (interleukin-23), IL-10, and IFN-beta, that eventually promote an immune response against infection (65, 66). In the case of the proposed construct-IV, significantly elevated levels of cytokines and interleukins, including IFN-γ and TGF-B, were predicted after continuous antigen exposure during immunization periods, while the other cytokines present lower concentration detection (**Figure 9I**). The Simpson’s Index (D) was measured normal, indicating that vaccine-V1 has an analytically broader impact (67). These immune simulation predictions suggest that the prioritized vaccine-V1 has the potential to activate T and B cells to produce antibodies, leading to the development of long-lasting memory cells after repeated antigen exposure. The immune simulation prediction suggested that the vaccine construct V1 holds the potential to induce strong innate and adaptive immune responses in the human immune system against leishmaniasis.

### 3.11 Codon optimization and *in silico* restriction cloning

**Figure 10:**
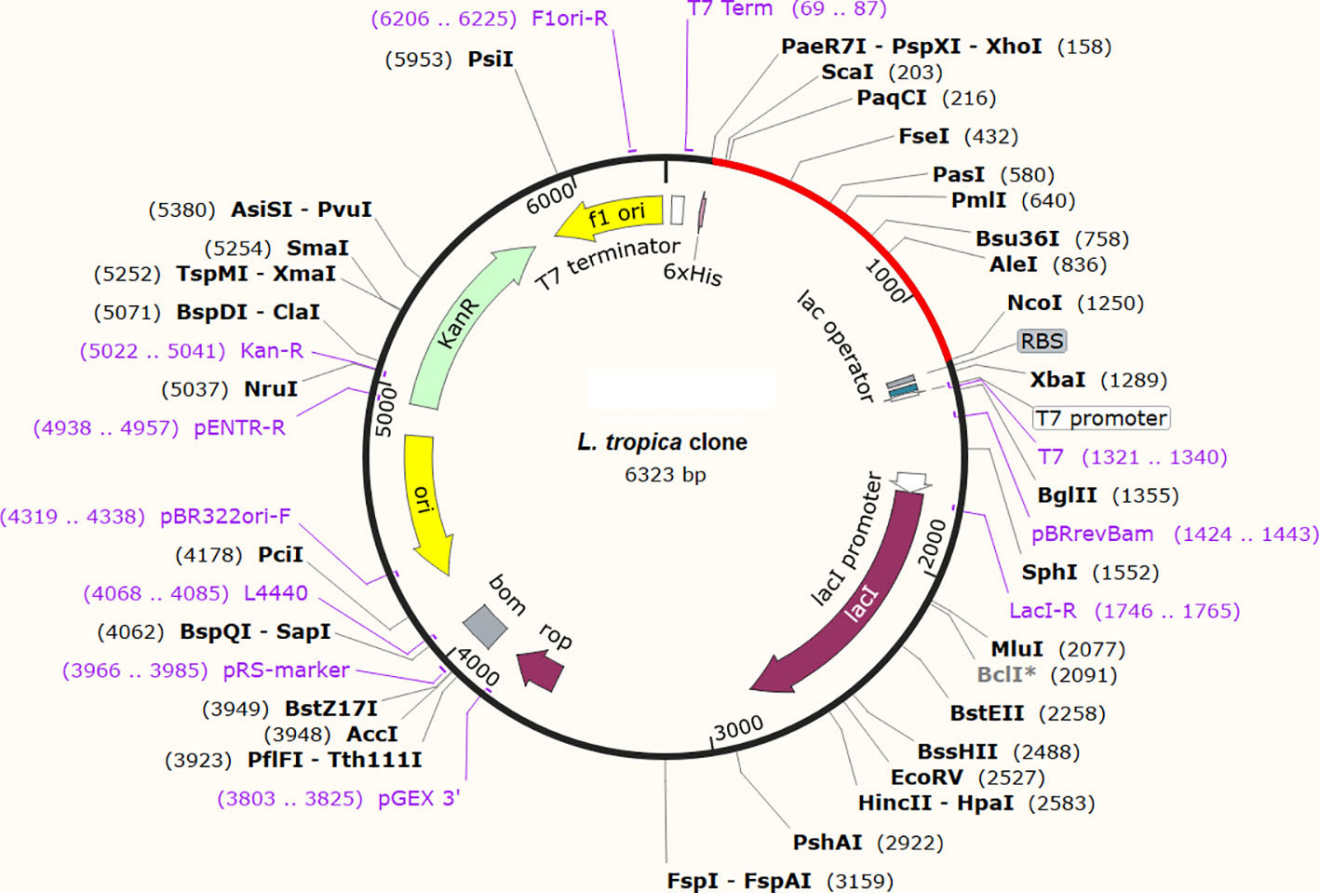
*In-silico* restriction cloning of the optimized gene sequence of the finalized vaccine construct in *E. coli* vector. The red color indicates the sequence of the vaccine and the black color indicates the sequence of the plasmid.

The expression potential of the proposed constructs was examined. The JCAT results for the optimized cDNA predicted that all the proposed vaccine constructs exhibited CIA values of 1.0 and GC content of 48-51%, which are within the optimal range of favorable expression of vaccine constructs in the *E. coli* K12 vector (**Table 4**) (68). The optimized gene sequence of the prioritized vaccine construct was predicted to successfully clone in the commonly used pET128 (+) plasmid with a total length of 6323 bp (**Figure 10**).

## 4 Discussion

*L. tropica* is one of the major causes of leishmaniasis, affecting millions of individuals worldwide. The World Health Organization (WHO) has listed leishmaniasis as one of the most neglected tropical diseases (6). Therefore, the development of novel treatment strategies is critical for providing protection against leishmaniasis. The availability of a potent vaccine against leishmaniasis is the most efficient and cost-effective approach to reduce mortality and morbidity. Advances in next-generation sequencing technology and the availability of massive genomic and proteomic data in public databases have led to the development of novel approaches, including immunoinformatics and reverse vaccinology, for identifying novel vaccine candidates against life-threatening pathogens in a cost- and time-effective manner (69, 70). Multiple preclinical trials were conducted to evaluate numerous vaccine candidates against leishmaniasis; however, a few have advanced to the clinical trial stage (71). Currently, no commercial vaccine is available against leishmaniasis. Previous studies targeted *L. major* and *L. infantum* to map potential epitopes against these pathogens (72). Likewise, a multi-epitope vaccine previously proposed against *L. donovani*, which also causes leishmaniasis (73). The present study aimed to design a next-generation multi-epitope chimeric vaccine against leishmaniasis that is capable of eliciting the human immune system and generating innate and adaptive immunity against *L. trotpica-*mediated infection.

In this study, we prioritized five extracellular *L. tropica* proteins as vaccine candidates. These proteins were non-homologous to human and human gut proteomes, as well as reported to be essential for the survival of the parasite. These proteins are predicted to be located in the extracellular regions and possibly be the first molecules to interact with the host cells; hence, making them ideal vaccine candidates to target (74). Stringent criteria were followed to identify CTL, HTL, and linear B-cell epitopes. B-cells induce humoral immune responses that neutralize pathogenic reagents and establish a memory to defend against future exposures (75). T cells, i.e., CTLs and HTLs, trigger cellular immune responses that prevent the spread of disease by eradicating infected cells or by secreting anti-microbial cytokines that provide long-lasting immunity for decades (76). B- and T-cell epitopes were further prioritized based on antigenicity, non-allergenic, and non-toxic parameters. The selected epitopes covered approximately 80% of the global population. Overlapping lead MHC-I, MHC-II, and B-cell epitopes in combination with specific linker and adjuvant sequences were used to design multi-epitope-based chimeric vaccine constructs. Similar *in-silico* strategies have been used to design vaccine constructs against multiple pathogens, including *Acinetobacter baumannii, Salmonella Typhimurium, Trypanosoma vivax*, COVID-19, Ebola virus, and Marburg, which have been validated experimentally (77–81). The designed vaccine models in this study are predicted to exhibit high antigenicity as well as low allergenicity and toxicity. The predicted physicochemical properties proposed that the vaccine constructs were highly stable with enhanced hydrophilicity, suggesting their ability to induce strong immunogenic responses in the human immune system. Thermodynamic stability, small molecular weight, and solubility predicted that the vaccine construct could easily be produced and administered in the host.

The 3D structural information is crucial to examine the activity of the vaccine by understanding the biomolecular interactions of the proposed vaccine to human immune cell receptor molecules. The tertiary structural prediction and validation of the proposed vaccines were carried out using multiple computational tools. The proposed vaccine constructs were significantly improved and displayed desirable characteristics in terms of quality and stability as predicted by Ramachandran plot values and Z-scores. Previous studies reported that human TLR receptors have been associated with the identification of pathogenic peptides and are responsible for the stimulation of immune responses against specific pathogens (82). Therefore, the molecular docking analysis of *L. tropica* vaccines was carried out against human TLR4 receptors. The docking results determined strong binding interactions between vaccine-TLR4 complexes. Vaccine construct-V1 showed the lowest binding energy with the human TLR4 receptor. The thermodynamic stability of the V1-TLR4 complex was verified by binding affinity, normal mode dynamics, and immune simulations analyses (83–86). Immunogenic responses of TLR4 have been reported previously to inhibit parasite load and reduce inflammation in CL (82). Immune simulations indicated regular and effective immune responses. Stronger immune responses were triggered as a result of repeated exposure to the antigenic vaccine construct-V1. Helper T cells were stimulated and memory B and T cells were found to develop. Strong Ig and Th-cell production enhanced the humoral immune response. A similar study reported the same immune patterns for various pathogens (78, 79, 87). The C-ImmSim application prediction has been validated experimentally in recent studies that reported more or less the same immunization pattern against viral antigens as predicted by the resource (88, 89). The results suggested that the prioritized vaccine construct-V1 holds significant potential to activate human TLR receptors and trigger both humoral and cell-mediated immune responses against leishmaniasis. The computational restriction cloning of the vaccine cDNA sequence in an *E. coli* plasmid ensured the expression capability of the vaccine construct-IV in the bacterial expression system. All the analyses pursued lay a framework for the development of an effective anti-leishmania vaccine capable of eliciting significant immunological responses in the human host immune system.

The current study presents a multi-epitope chimeric vaccine design utilizing the *L. tropica* protein components, which is one way to deal with antigenic complexities. However, the current study holds some limitations. Immunoinformatics-based vaccine construction depends heavily on prediction methods. The degree of protection against *L. tropica* infection is unclear, and the accuracy of these prediction approaches may be limited. Standard benchmarking, restricted prediction methodologies, and a lack of accurate datasets for diverse computational studies are only a few of the difficulties associated with immunoinformatics-based methods. Although immunoinformatics predictions have led to some positive case reports in recent years (90–92), the findings of this study still need to be investigated through *in vitro* and *in vivo* bioassays to confirm the safety and effectiveness of the proposed vaccine against leishmaniasis.

## 5 Conclusion

The current work employs subtractive proteomics and reverse vaccinology strategies to generate a novel multi-epitope chimeric vaccine construct against *L. tropica*. Extracellular proteins were used to identify B- and T-cell epitopes. Lead overlapping B- and T-cell epitopes were prioritized based on antigenicity, allergenicity, and toxicity characteristics to create a unique multi-epitope vaccine construct. The immunological features predicted the prioritized vaccine models to be non-allergenic, non-toxic, and highly antigenic. The physiochemical features predicted structure stability, and high solubility of the proposed vaccine models. Molecular docking, normal mode analysis, and molecular dynamic simulations established the stability and strong binding interactions of the top-ranked vaccine construct with human immune receptors. Immunological simulation revealed that the proposed vaccine design is capable of eliciting cell-mediated and humoral immune responses in the host immune system. The findings of this study needs to be validated experimentally.

## Data availability statement

The original contributions presented in the study are included in the article/**Supplementary Material**. Further inquiries can be directed to the corresponding authors.

## Author contributions

SA: Formal Analysis, Investigation, Methodology, Writing – original draft, Writing – review & editing. AA: Data curation, Formal Analysis, Writing – original draft, Writing – review & editing. AAK: Methodology, Writing – review & editing. AM: Writing – review & editing. AS: Writing – review & editing. SA: Formal Analysis, Writing – review & editing. CL: Writing – review & editing. ZR: Funding acquisition, Supervision, Writing – review & editing. AK: Data curation, Formal Analysis, Writing – review & editing. SK: Writing – review & editing, Supervision, Writing – original draft.

## Supplementary material

Supplementary File 1 multi-fasta file containing non-paralogous proteins from *L. tropica*;

Supplementary File 2 multi-fasta file of parasite essential proteins;

Supplementary File 3 parasite essential proteins that are non-homologous to human and human gut microbiota.

Supplementary Data Sheet all four designed vaccine constructs.
