# Supplementary material for: Vaccinomics-based next-generation multi-epitope chimeric vaccine models prediction against Leishmania tropica - a hierarchical subtractive proteomics and immunoinformatics approach
Source: Front Immunol. 2023 Sep 15;14:1259612. doi: 10.3389/fimmu.2023.1259612 (PMC10540849; doi:10.3389/fimmu.2023.1259612)
Supplement: Supplementary Data Sheet — all four designed vaccine constructs. [file DataSheet_2.pdf]

Construct #1 adjuvant = HBHA adjuvant

EAAAKMAENPNIDDLPAPLLAALGAADLALATVNDLIANLRERAEETRAETRTRVEERRARLTKFQEDLPEQFIELRDKFT
TEELRKAAEGYLEAATNRYNELVERGEAALQRLRSQTAFEDASARAEGYVDQAVELTQEALGTVASQTRAVGERAAKLV
GIELEAAAKAKFVAAWTLKAAAGGGSMSLPNWSANRQSIGARAGPAGGGSYTLDAAVCVPFGGSGADASRGGGSFF
TPFGVTAGTESDLMASALGGGSRASSSNVTPINYSAHVVPSQGGGSTLKRDERDDASGRDTRNLSFGGGSSRTQIPLRH
AWALTIHKSQGGGGSNGGAPSSTSVATVYASPTQAGGGSPTPTQMLTPQANAAAAAAAVGGGSAKFVAAWTLKAA
AGGGSYVAAPQDTGRASVGVEHRVIHEYGAEALERAGVVSNELAVSHKTCGREEAATHEYGAEALERAGAKFVAAWT
LKAAAGGGS

Construct #2 adjuvant = Beta definsin adjuvant

EAAAKGIINTLQKYYCRVRGGRCAVLSCLPKEEQIGKCSTRGRKCCRRKKEAAAKAKFVAAWTLKAAAGGGSMSLPNW
SANRQSIGARAGPAGGGSYTLDAAVCVPFGGSGADASRGGGSFFTPFGVTAGTESDLMASALGGGSRASSSNVTPINY
SAHVVPSQGGGSTLKRDERDDASGRDTRNLSFGGGSSRTQIPLRHAWALTIHKSQGGGGSNGGAPSSTSVATVYASPT
QAGGGSPTPTQMLTPQANAAAAAAAVGGGSAKFVAAWTLKAAAGGGSYVAAPQDTGRASVGVEHRVIHEYGAEAL
ERAGVVSNELAVSHKTCGREEAATHEYGAEALERAGAKFVAAWTLKAAAGGGS

Construct #3 adjuvant= HBHA conserved adjuvant

EAAAKMAENSNIDDIKAPLLAALGAADLALATVNELITNLRERAEETRRSRVEESRARLTKLQEDLPEQLTELREKFTAEEL
RKAAEGYLEAATSELVERGEAALERLRSQQSFEEVSARAEGYVDQAVELTQEALGTVASQVEGRAAKLVGIELEAAAKA
KFVAAWTLKAAAGGGSMSLPNWSANRQSIGARAGPAGGGSYTLDAAVCVPFGGSGADASRGGGSFFTPFGVTAGTE
SDLMASALGGGSRASSSNVTPINYSAHVVPSQGGGSTLKRDERDDASGRDTRNLSFGGGSSRTQIPLRHAWALTIHKS
QGGGGSNGGAPSSTSVATVYASPTQAGGGSPTPTQMLTPQANAAAAAAAVGGGSAKFVAAWTLKAAAGGGSYVA
APQDTGRASVGVEHRVIHEYGAEALERAGVVSNELAVSHKTCGREEAATHEYGAEALERAGAKFVAAWTLKAAAGGG
S

Construct #4 adjuvant = Ribosomal protein adjuvant

EAAAKMAKLSTDELLDAFKEMTLLELSDFVKKFEETFEVTAAAPVAVAAAGAAPAGAAVEAAEEQSEFDVILEAAGDKKI
GVIKVVREIVSGLGLKEAKDLVDGAPKPLLEKVAKEAADEAKAKLEAAGATVTVKEAAAKAKFVAAWTLKAAAGGGSM
SLPNWSANRQSIGARAGPAGGGSYTLDAAVCVPFGGSGADASRGGGSFFTPFGVTAGTESDLMASALGGGSRASSSN
VTPINYSAHVVPSQGGGSTLKRDERDDASGRDTRNLSFGGGSSRTQIPLRHAWALTIHKSQGGGGSNGGAPSSTSVAT
VYASPTQAGGGSPTPTQMLTPQANAAAAAAAVGGGSAKFVAAWTLKAAAGGGSYVAAPQDTGRASVGVEHRVIHE
YGAEALERAGVVSNELAVSHKTCGREEAATHEYGAEALERAGAKFVAAWTLKAAAGGGS

Blue color – EAAAK linker to join adjuvant with vaccine construct

Black color – adjuvant sequences

Red color – epitopes

Grey color – GGGS and HEYGAEALERAG linkers to connect epitopes

Green color – PADRE sequences
